# Cocreation in Health Workforce Planning to Shape the Future of the Health Care System in the Philippines

**DOI:** 10.9745/GHSP-D-22-00176

**Published:** 2022-12-21

**Authors:** Harvy Joy Liwanag, Jhanna Uy, Mary Ruth Politico, Mary Joy Padilla, Ma. Catherine Arzobal, Kaycee Manuel, Angeli Loren Cagouia, Pretchell Tolentino, Annika Frahsa, Kenneth Ronquillo

**Affiliations:** a Institute of Social and Preventive Medicine, University of Bern, Bern, Switzerland.; b Balik Scientist Program, Department of Science and Technology, Philippine Council for Health Research and Development, Taguig, Philippines.; c Philippine Institute for Development Studies, Quezon City, Philippines.; d Health Sciences Program, School of Science and Engineering, Ateneo de Manila University, Quezon City, Philippines.; e Health Human Resource Development Bureau, Department of Health, Manila, Philippines.

## Abstract

**Background:**

The Philippines passed landmark legislation in 2019 on universal health coverage, including reforms in the development of its health workforce, an essential building block of responsive health care systems.

**Health Workforce Planning Cocreation Process:**

We based our planning process on a model of cocreation defined as sharing power and decision making to solve problems collaboratively and build consensus around action. Through cocreation with policy makers, researchers, and other stakeholders, we performed projection studies on 10 selected health professions and estimated the need for primary care at national and subnational levels, which was the most extensive health workforce projection carried out by the Philippine Department of Health to date. We determined health workforce requirements based on target densities recommended by the World Health Organization and a health needs approach that considered epidemiological and sociodemographic factors. In consultation with stakeholders, we interpreted our analysis to guide recommendations to address issues related to health workforce quantity, skill mix, and distribution. These included a broad range of proposals, including task shifting, expanding scholarships and deployment, reforming health professionals’ education, and pursuing a whole-of-society approach, which together informed the National Human Resources for Health Master Plan.

**Conclusions:**

Our cocreation model offers lessons for policy makers, program managers, and researchers in low- and middle-income countries who deal with health workforce challenges. Cocreation led to relationship building between policy makers and researchers who jointly performed the research and identified solutions through open communication and agile coordination. To shape future health care systems that are responsive both during normal times and during crises, cocreation would be essential for evidence-informed policy development and policy-relevant research.

## Abstract (precis)

Cocreation in health workforce planning in the Philippines led to relationship building between policy makers and researchers who jointly identified solutions to address challenges in the health care system.

## Teaser

**Key Messages:**

- The COVID-19 pandemic presented strategic opportunities to strengthen national health workforce planning to shape future health care systems that are responsive both during normal times and during crises.
- We describe our health workforce planning process based on a cocreation model that built a relationship between researchers and policy makers to coproduce recommendations to strengthen primary care and advance universal health coverage in the Philippines.
- We applied 2 approaches to project the future supply of 10 selected health professions and estimated the demand for primary care services at national and subnational levels; our analysis provided spaces for policy recommendations on issues related to health workforce quantity, skill mix, and distribution.

**Key Implication:**

- Lessons from our experience may guide policy makers, program managers, and researchers in low- and middle-income countries as they navigate the challenges in their health workforce through cocreation where the research addresses policy-relevant questions and the resulting policy is informed by the research evidence.

## BACKGROUND

The failure to prevent the scale of the coronavirus disease (COVID-19) pandemic derived less from countries’ lack of preparedness than from an inability to act effectively on plans despite many years of warnings.^1^ Planning should include the development of the health workforce—an important building block of responsive health care systems.^2^ An adequate and competent health workforce during normal times would set the stage for responsive and resilient health care systems during crises, such as the COVID-19 pandemic.^3^ Although the pandemic was unprecedented in its scale, the challenges it posed could have been mitigated by a cocreation approach to health workforce planning and a foresight perspective to shape the future of health care systems. In the literature, there are several examples of conventional health workforce planning,^4^ but little has been written about how cocreation could facilitate the planning process and open spaces for collaborative action.

In this program case study, we describe our cocreation process to generate and analyze data on health professions as part of strategic planning to inform the development of a national health workforce master plan for the Philippines. By describing both our process and findings, we seek to provide relevant lessons for policy makers, program managers, researchers, and other stakeholders who deal with health workforce issues in low- and middle-income countries.

### Toward Universal Health Coverage Through Primary Health Care

The need for an updated health workforce national plan for the Philippines was prompted by the passage in July 2019 of landmark legislation on universal health coverage (UHC), which guaranteed UHC for all Filipinos and prescribed complementary reforms in the health care system.^5^ The World Health Organization (WHO) has defined UHC as all people receiving quality health services that meet their needs without exposure to financial hardship.^6^ The comprehensive primary health care approach, instituted in the 1978 Alma-Ata declaration, has been widely recognized as the cornerstone of a responsive health care system to accelerate UHC.^7^ Primary care, the service provision element of primary health care,^8^ was envisioned in the Philippine UHC law as “initial contact, continuous, comprehensive, and coordinated care accessible at the time of need, with a range of services for all presenting conditions, and able to coordinate referrals to other health care providers.”^5^ Despite several national policies and reforms undertaken in the past 30 years since the devolution of the public health sector,^9^ pri-mary care has remained weak in the Philippines, where the health care system is a mix of public and private sectors that run parallel at various levels, driven by free-market forces that result in higher costs, barriers to care, and health care system inefficiencies.^10^ Patients could directly access specialist care even for simple conditions, a common practice also shaped by a health professions education system that has prioritized specialization over primary care practice.^10^ Reorienting health professions’ education toward training a health workforce that is competent in primary care is one of the complementary reforms enshrined in the Philippine UHC law.^5^

The need for an updated health workforce national plan for the Philippines was prompted by the passage of landmark legislation on UHC, which guaranteed UHC for all Filipinos and prescribed complementary reforms in the health care system.

### A Need for Cocreation to Bridge Research and Policy

UHC cannot exist without an adequate, competent, highly motivated, and equitably distributed health workforce that can deliver primary care services effectively to meet the needs of the people.^11^ However, health workforce supply and distribution are particularly challenging in an archipelago like the Philippines, which is composed of about 7,640 islands and a population of 110 million people.

The Philippine Department of Health (DOH) has been mandated by the UHC law to develop the National Human Resources for Health Master Plan^12^ to strengthen the delivery of primary care, accelerate UHC, and shape the future of the health care system. The plan provided policies and strategies for the “appropriate generation, recruitment, retraining, regulation, retention, and reassessment of the health workforce based on population health needs.”^5^ The implementing rules and regulations^13^ of the UHC law identified staffing requirement standards responsive to changing population health needs as a key component of the master plan. Therefore, the plan contained strategies that drew on evidence while also ensuring close collaboration among stakeholders in the health care system. Although the DOH and its Health Human Resource Development Bureau (HHRDB)^14^ had extensive institutional experience in developing health workforce policies at the national level, in-house expertise in research methods and data analysis was limited. Previous workforce planning exercises have often been conducted through external partners or consultants based on a conventional model of knowledge translation. However, there is growing recognition of using cocreation to develop solutions collaboratively.

**FIGURE 1:**
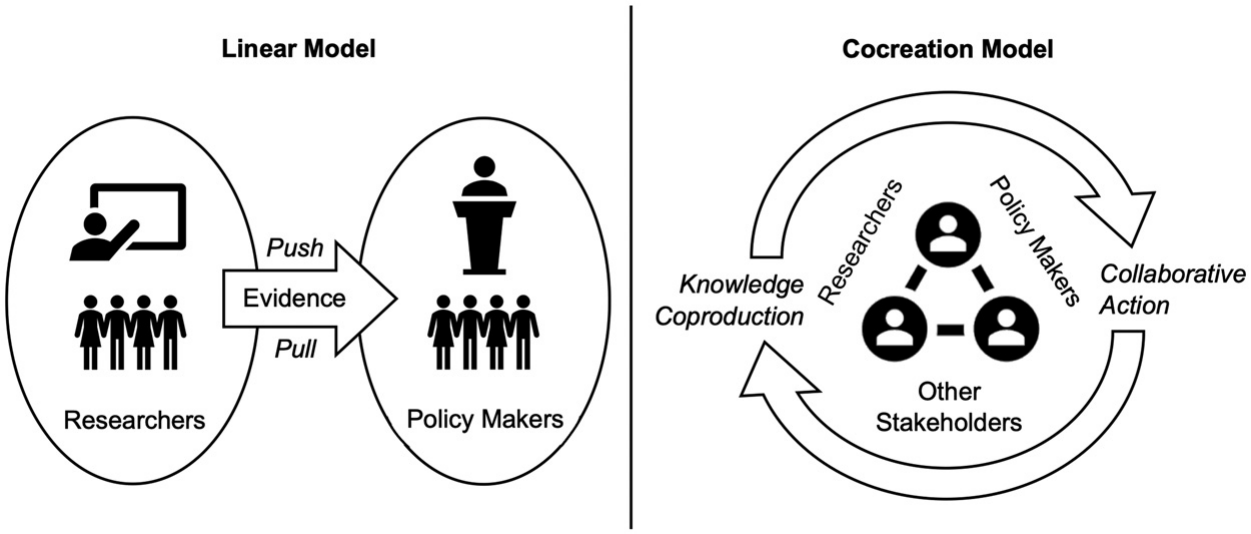
The Linear Model of Research Translation Versus the Cocreation Model Used by the Philippine Department of Health in the Health Workforce Planning Process

Different definitions and models of cocreation stem from diverse disciplines such as business studies, design science, computer science, community development, and health research.^15^ Briefly, cocreation is often described as collaborative knowledge generation that moves beyond academia and works closely with stakeholders to achieve societal impact.^15^ Within our case study, we referred to cocreation as sharing power and decision making by bringing stakeholders together to identify problems, design solutions collaboratively, and build consensus around action.^16^ Through our cocreation process, researchers worked closely with policy makers to perform data analysis and interpretation to address policy questions for the health workforce, while policy makers worked with the researchers to use the evidence for health workforce planning and policy. Hence, cocreation provided a platform that integrated the domains of research, policy, and practice^17^ where stakeholders engaged one another in the process. It is a departure from the linear push-and-pull models of research utilization, which is more commonly practiced in the Philippines and most other countries, in favor of an interactive and dynamic model of knowledge coproduction with seamless translation into collaborative action.^18^ Figure 1 illustrates our application of cocreation for health workforce planning in contrast to the linear model of research translation.

Cocreation provided a platform that integrated the domains of research, policy, and practice where stakeholders engaged one another in the process.

## COCREATION PROCESS FOR HEALTH WORKFORCE PLANNING

In the following sections, we describe the initial phases of cocreation when we performed planning for the health workforce in the Philippines.

### Researchers Initiated the Process

The research team initiated the process by approaching policy makers and program managers in the DOH to explore areas for cooperation. A series of initial discussions focused on the expertise of the research team members, who were trained overseas, and the priorities of the DOH. The HHRDB team in DOH identified health workforce projections as a priority area for convergence to inform the development of the master plan. The research team proposed the extent of expertise available that could be tapped to support the studies.

### Financing Instrument and Partnering Arrangement Facilitated the Process

The research team explored domestic financing instruments that could support the costs of labor and research activities to perform the studies. The team identified the Balik Scientist Program (BSP)^19^ of the Philippine Department of Science and Technology (DOST) as a suitable financing and capacity-building mechanism as it enables Filipino scientists who trained and/or resided abroad to share their expertise with local host institutions and contribute to scientific capacity strengthening. In the past, Filipino scientists who were engaged through the BSP collaborated mostly with academic institutions. The research team and the DOH leveraged the BSP for a creative collaboration in which the researchers were hosted instead in a nonacademic setting to bridge the gap between research and policy and practice. The collaboration was formalized through a tripartite agreement signed by the research team and high-level officials of the DOST and DOH through which DOST financed the activities of the collaboration, the DOH identified the policy questions and hosted the research team during the engagement, and the research team led the research together with the HHRDB team in the DOH.

### Researchers and Policy Makers Shared Resources

The researchers used a workspace provided by the DOH within its headquarters, and the technical staff in the DOH shared access to government-managed data. The arrangement provided an environment for building relationships between the researchers and DOH staff composed of junior, midlevel, and senior program managers and policy makers. Daily interactions in the office led to better appreciation by the research team of the needs and perspectives of policy makers and program managers. Simultaneously, the DOH team gained a better understanding of research methods through regular sessions during which researchers conducted brief introductory seminars or workshops for the HHRDB team. The priority policy questions to be addressed by the health workforce projections were jointly formulated through these series of interactions.

Shared workspace allowed for daily interactions and sessions which contributed to better understanding between researchers, policy makers, and program managers.

### Guiding Questions Informed the Priorities

The research team identified and prioritized the following questions:

- Quantity: How do we assess the adequacy of the health workforce and meet current and future population demand for health services, particularly primary care?
- Skill mix: How do we achieve an appropriate mix of cadres of the health workforce?
- Distribution: How can the distribution of the health workforce be more equitable to reach the most underserved areas?

We expanded from these questions by further addressing 5 key questions on what, who, where, when, and why that guided how we proceeded with our choice of methods to determine health workforce requirements.

- What? Projection studies on the future supply of the health workforce would draw on past and current trends in the production of health professionals in the Philippines and would assess whether the supply could match future demand for health services.
- Who? Projection studies would focus on the set of cadres considered by the DOH team as essential in the delivery of primary care services. These included a total of 10 health professions: physicians, nurses, midwives, dentists, registered medical technologists, radiologic technologists, pharmacists, nutritionist-dieticians, physical therapists, and occupational therapists. These cadres were selected because their health professional associations had also been participating in the broader Human Resource for Health Network (HRHN), a multistakeholder body of 18 governmental and nongovernmental organizations convened by the DOH that meets regularly to harmonize policy directions and coordinate efforts toward addressing health workforce challenges.^20^
- Where? Projections would be performed at the national level of analysis to inform the national health workforce master plan but would also explore health workforce requirements at subnational levels to guide actions at local levels.
- When? Analyses would draw on data on the number of licensed health professionals covering at least a 10-year period (2010–2020), would be complemented by additional information on epidemiological and sociodemographic factors, and would project health workforce requirements until 2050.
- Why? Projecting health workforce requirements would place the Philippine government in a better position to intervene to address anticipated gaps in service provision and strategically shape the future development of a health workforce.

Guided by these 5 questions, we projected health workforce requirements for the Philippines to address the questions related to quantity, skill mix, and distribution. We reviewed the spectrum of approaches^4^ to determine health workforce requirements. The simplest tools (e.g., workforce-to-population thresholds) would require fewer data and resources but would be less sensitive to changing population health needs. In contrast, the more sophisticated tools could capture health demand more accurately but would require additional data and resources. Without any approach to assess health workforce requirements as part of the planning process, health workforce quantity, skill mix, and distribution would be left to the influence of market forces, which would aggravate health inequity and hinder UHC. We compare the various assessment approaches in Table 1.^21^^–^^24^

**TABLE 1.** Approaches to Assess Health Workforce Requirements

	**Approach**	**Defining Workforce Requirements**	**Advantages**	**Disadvantages**	**Examples**
Less data required, but less sensitive to changing population health needs	Workforce-to-population ratios	Benchmarks of workforce density	Straightforward and less data required	Insensitive to changing population health needs	WHO workforce requirements for UHC^21^
	Service utilization/ service target approach	Current levels of service utilization, or future targets in service delivery	Simple but requires availability of data on service utilization	Utilization patterns not necessarily an indicator of met demand	Thailand study by Pagaiya et al.^22^
More data and resources required, but more accurate in estimating need	Health needs approach	Sociodemographic and epidemiological data	More accurate assessment of health needs based on disease burden	Requires more data (e.g., burden of disease) which may not be readily available	Mental health workforce in LMICs^23^
	Workload Indicators of Staffing Need	Workload pressure in a health facility against activity standards	Provides a strong basis for health workforce needs up to health facility level	Requires additional capacity and time to perform data collection and analysis	Philippines study by Aytona et al.^24^
	No approach to guide planning; market forces determine health workforce quantity, skill mix, and distribution.				

Abbreviations: LMICs, low- and middle-income countries; UHC, universal health coverage; WHO, World Health Organization.

We projected health workforce requirements for the Philippines to address the questions related to quantity, skill mix, and distribution.

The WHO has promoted the Workload Indicators of Staffing Need (WISN) as a systematic approach to guide staffing decisions based on health workers’ workload in a given facility against activity time standards.^25^ The DOH previously piloted the use of WISN to assess health workforce shortages in selected regions in the Philippines.^24^ Although considered to be a comprehensive tool, WISN would require the training of staff for data collection and analysis and additional resources for national scale-up.

The research and DOH teams jointly deliberated the advantages and disadvantages of each approach and considered the trade-offs to determine the most suitable methodology that was (1) accurate enough to project the future numbers of the health workforce and the future need for health services; (2) based on information already accessible to the DOH without the need for further data collection requiring additional time and resources; (3) feasible given the technical expertise available among stakeholders in the cocreation process; and (4) likely to be completed to meet the DOH deadline of 2021 to publish the master plan. We decided to use the approach based on workforce-to-population ratios as we considered it the simplest tool that could produce early results to guide our decision making. However, we adopted a combination of approaches by complementing it with a health needs approach that considered burden of disease and sociodemographic data.

We adopted a combination of approaches, complementing the use of workforce-to-population ratios with a health needs approach.

### Projection Studies Estimated The Future Workforce Needs

We engaged the Professional Regulation Commission (PRC) to obtain health professions data. Through a formal interagency agreement on data sharing, PRC provided the DOH with access to the dataset containing numbers of licensed health professionals in the Philippines since 2010. We analyzed the previous numbers of licensed health professionals and projected the future numbers of health professionals in 2025 and every 5 years thereafter until 2050. Projections were a straightforward application of the Leslie model^26^ based on stable population theory under the assumption that production and attrition of health professionals by age group would be stable over time. Projections for each selected health profession in subsequent periods were the combined numbers of (1) new entrants, captured by health professionals’ reproduction rate calculated from the PRC dataset and the 2010 census-based population projections^27^; and (2) the remaining health professionals from the previous period, captured by attrition/hazard rate and base stock.

#### Establishing Benchmarks to Achieve UHC and the Sustainable Development Goals

We referred to the WHO-recommended threshold of 44.5 skilled health workers (i.e., physicians, nurses, and midwives) per 10,000 people as the minimum health workforce density to achieve UHC and the Sustainable Development Goals (SDGs).^21^ However, we wanted to determine the share of each health profession instead of a combined density. Therefore, we referred to the WHO benchmark of 30.2 nurses and midwives per 10,000 people to achieve UHC and the SDGs, where the target density specific for nurses is 27.4.^28^ Then, we calculated the threshold specific for midwives as 2.8 (i.e., 30.2 minus 27.4) and that for physicians as 14.3 (i.e., 44.5 minus 30.2). We had no reference for the benchmarks for the 7 other health professions included in our projections as the WHO has not recommended thresholds for these cadres.

**FIGURE 2:**
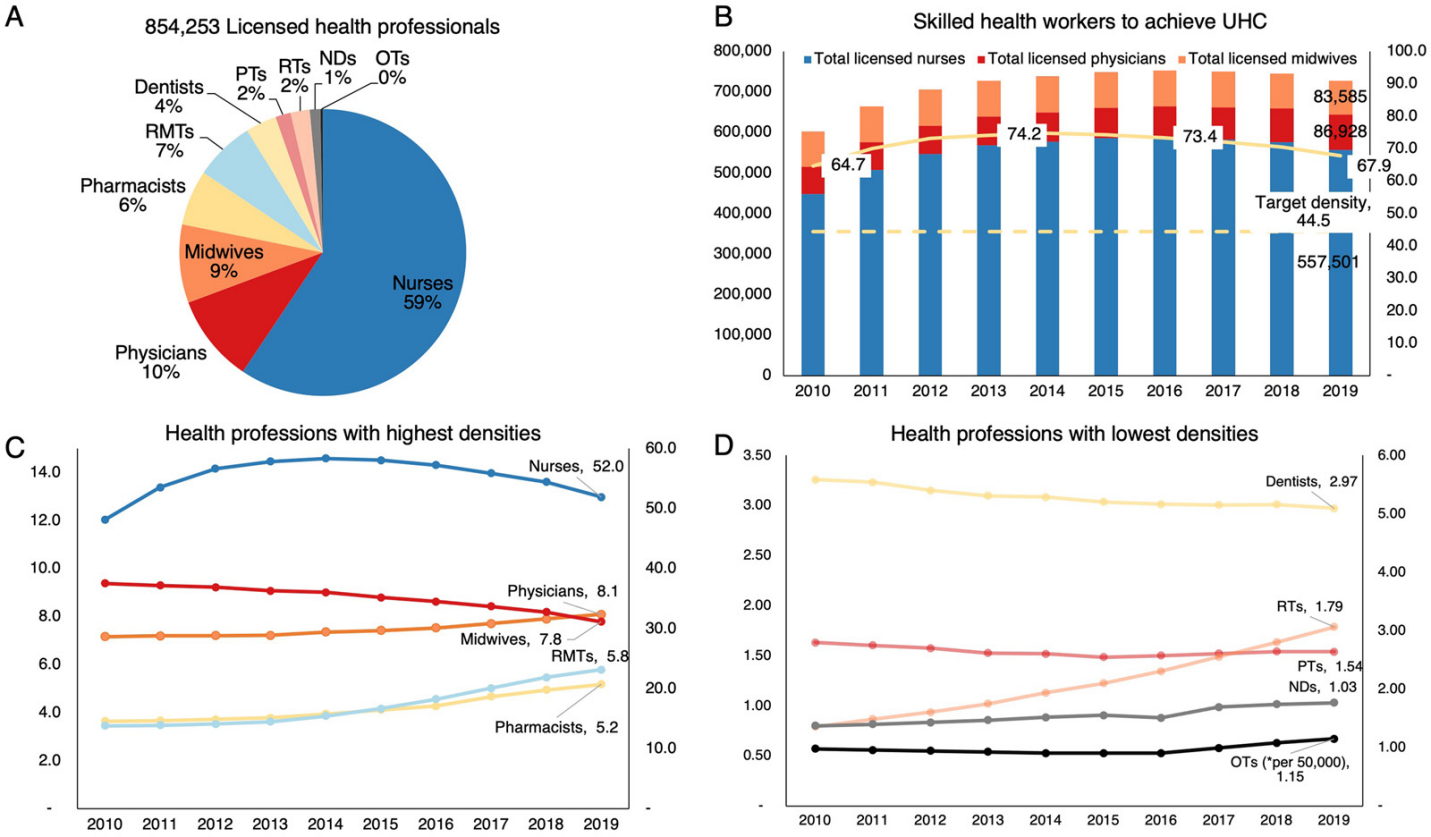
Total Number of Licensed Health Professionals in the Philippines per Cadre, April 2020 (A); Density of Skilled Health Workers to Achieve UHC, by Cadre, 2010–2019 (B); Health Professions With the Highest Densities per 10,000 Population, 2010–2019 (C); Health Professions With the Lowest Densities per 10,000 Population,^a^ 2010–2019 (D) Abbreviations: NDs, nutritionist-dieticians; OTs, occupational therapists; PTs, physical therapists; RMTs, registered medical technologists; RTs, radiologic technologists; UHC, universal health coverage. ^a^ Per 50,000 for OTs from 2010 to 2019.

There was a total number of 854,253 licensed health professionals in the Philippines in April 2020, which was the time period when the dataset from the PRC was processed and analyzed (Figure 2). A majority (59%) of these licensed health professionals were nurses. This figure was consistent with the global trend in 172 countries where 59% of health professionals were nurses but was lower than the 68% average proportion in the WHO Western Pacific Region where the Philippines is situated.^28^

Combined densities for physicians, nurses, and midwives would suggest that the Philippines had exceeded the minimum threshold of 44.5 skilled health workers per 10,000 people to achieve UHC, reaching as high as 74.2 in 2013 and 67.9 in 2019. These data could create the illusion that the health workforce in the Philippines was already adequate to achieve UHC and that policy interventions to address health workforce shortages would no longer be necessary. When analyzed separately by health profession, the density of nurses (52 per 10,000) remained the highest among the health professions despite tapering in recent years. This high density of nurses could have diluted the combined density of skilled health workers, which appeared to have surpassed the threshold for UHC. In the last decade, there were also slight increases in the densities of physicians (8.1), medical technologists (5.8), pharmacists (5.2), and radiologic technologists (1.79). The densities of physical therapists (1.54 per 10,000), nutritionist-dieticians (1.03 per 10,000), and occupational therapists (1.15 per 50,000), which had the lowest density among the health professions, were relatively stable. In contrast, the densities of nurses, midwives (7.8 per 10,000), and dentists (2.97 per 10,000) were gradually declining in recent years.

**FIGURE 3:**
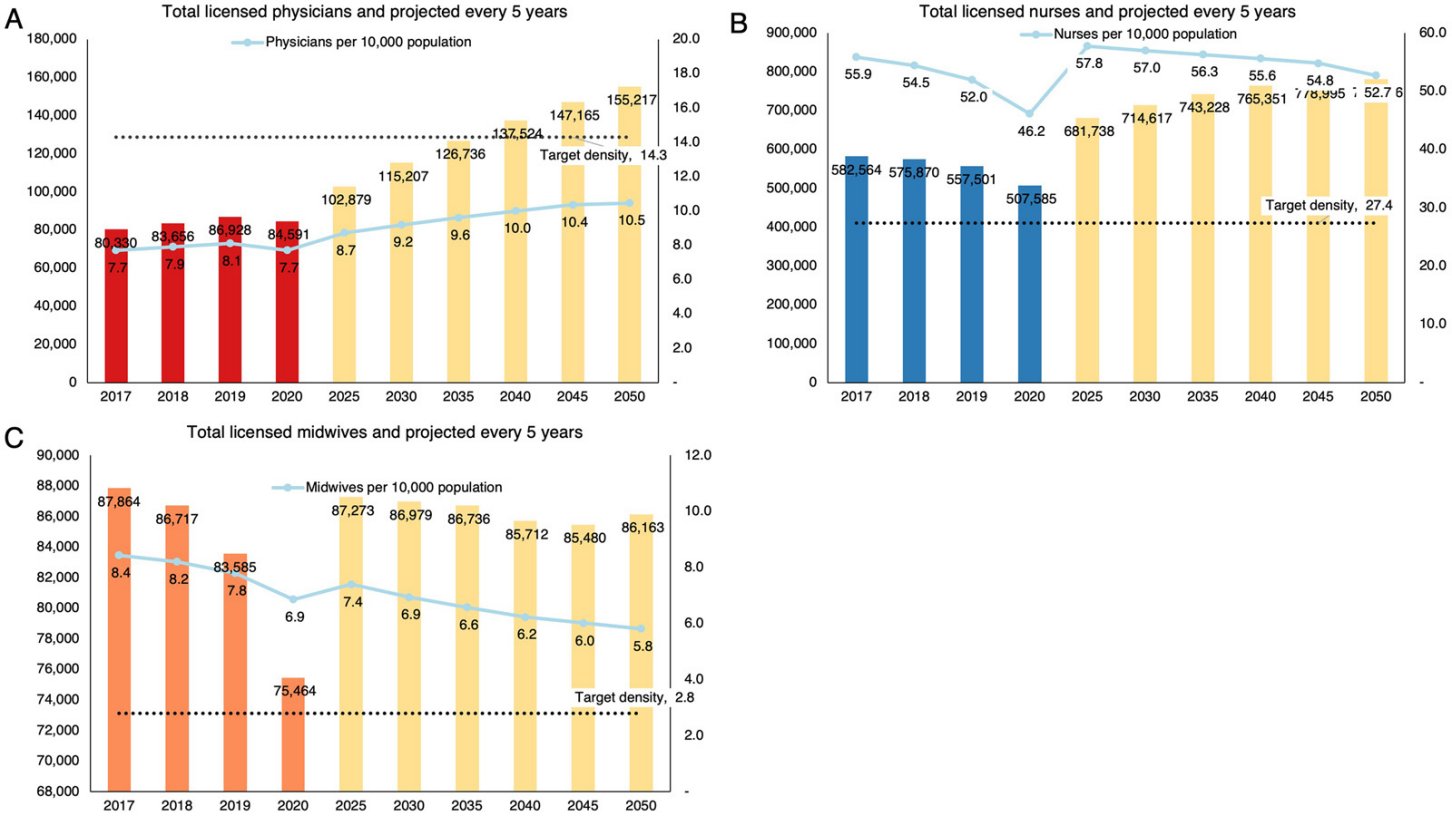
Number of Licensed Physicians (A), Nurses (B), and Midwives (C) in the Philippines per Year, 2017–2020, and the Projected Numbers and Densities Every 5 Years, 2025–2050, Against the Benchmark Thresholds for Universal Health Coverage^a^ ^a^ Numbers for 2020 were underestimated because of the licensure examinations delay caused by the coronavirus disease (COVID-19) pandemic.

Figure 3 illustrates the density of skilled health workers disaggregated according to physicians, nurses, and midwives. The ratio of 8.1 physicians per 10,000 people in 2019 was far below the target of 14.3, which would not be met despite the projected rise in the number of physicians from 2025 onward. The density of physicians in the Philippines was lower than the global density of 16.4 as well as the average density of 21 for the Western Pacific Region.^29^

For nurses, the density of 52 per 10,000 people in 2019 far exceeded the threshold of 27.4 and would continue to exceed the benchmark despite a projected tapering of the density beyond 2025. The density of nurses in the Philippines was significantly higher when compared to a similar archipelago like Indonesia (34.8),^30^ or Thailand (15.2) with a comparable economy,^31^ or Vietnam with a similar population size (14.5).^32^ The abundance of nurses in the Philippines has been attributed to overproduction^33^ by a nursing education system motivated by a labor export-oriented strategy that trains nurses primarily to migrate and work in high-income countries.^34^^,^^35^

Disaggregating the density of skilled health workers by cadre gave a more accurate assessment of the current workforce needs and gaps.

The ratio of 7.8 midwives per 10,000 people likewise surpassed the benchmark of 2.8 and would continue to be above the threshold in 2025 and beyond. The density of midwives in the Philippines was higher than the global density of 4.4, the density of 5.9 in the Western Pacific Region, and the average density of 4.3 in low- and middle-income countries.^36^ The shorter duration of 2 years to acquire a diploma in midwifery in the Philippines and qualify for licensure examinations^37^ could be a reason for the high number of midwives. Although the density of midwives surpassed the target ratio, the threshold assumes that skilled health workers would perform tasks well within their scopes of practice. The reality is that midwives would perform tasks beyond maternal, newborn, and child health and overlap with certain functions of other skilled health workers.^24^ A decline in the density of midwives would further widen the gap in service delivery as there would be less of this cadre that could assume some of the tasks in a primary care facility. Further studies are needed to probe the factors behind the projected decline in the densities of midwives as well as dentists to guide policy development for maintaining the densities of these health professions who play critical roles in maternal, newborn, and child health and oral health services.

**FIGURE 4:**
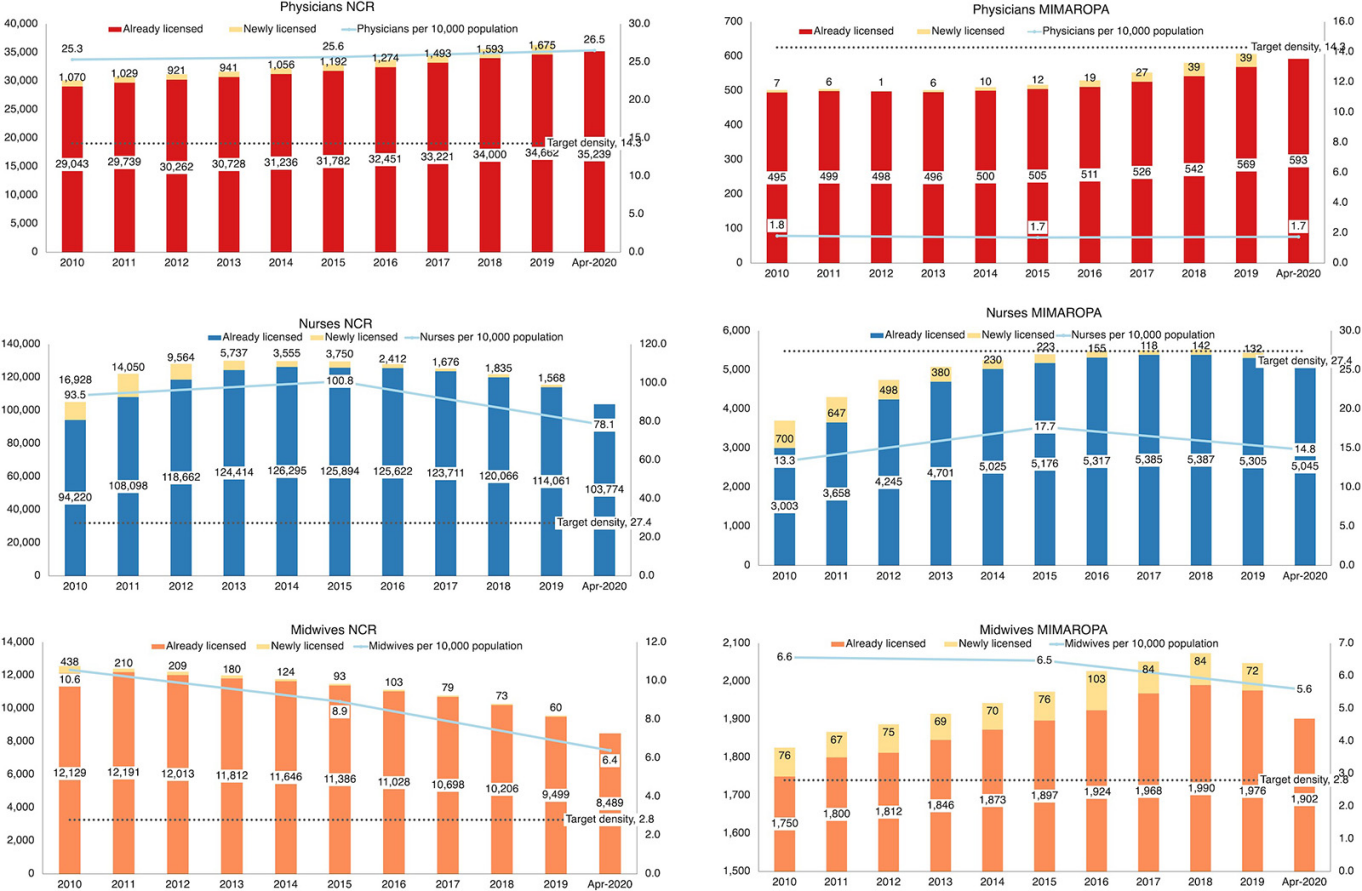
Number and Density of Licensed Physicians, Nurses, and Midwives in the Philippines, 2010–April 2020, Between the National Capital Region and Region IV-B in MIMAROPA Abbreviations: MIMAROPA, Mindoro, Marinduque, Romblon, and Palawan; NCR, National Capital Region.

Densities at subnational levels painted a different picture compared to the national situation (Figure 4). For example, in the National Capital Region, the country’s biggest metropolis that includes the capital of Manila, there were 26.5 physicians per 10,000 people in 2020, which far exceeded the target density of 14.3. On the other hand, the MIMAROPA island provinces region (Mindoro, Marinduque, Romblon, and Palawan) had a far lower density of physicians at 1.7 and also failed to meet the threshold for nurses at only 14.8 despite the abundance of nurses in the country. On the other hand, the threshold for midwives at 5.6 was above the benchmark. These densities contextualized our understanding of the inequitable distribution of the health workforce at subnational levels where certain regions had an abundance while other regions experienced severe shortages.

Calculating densities at subnational levels contextualized our understanding of the inequitable distribution of the health workforce.

#### Estimating Demand for Primary Care Services

We expanded our analysis by estimating the future demand for primary care services. To synergize efforts, we converged with a parallel initiative by the Health Facility Development Bureau in the DOH that estimated the gap in health infrastructure from primary to tertiary levels of care based on a health needs approach^4^ to develop a complementary Philippine Health Facility Development Plan.^38^ Drawing on the health needs approach as applied in estimating health infrastructure needs, we pegged future health workforce requirements on projected population needs for primary care services under the assumption that all needs should be met. Estimated need also considered the desired scenario of integrated health care provider networks with functional referral systems from primary to tertiary care and where need is met at the lowest level of care according to evidence-based guidelines.

We based the need for primary care services on the predicted prevalence and incidence rates of selected high-burden diseases for the period 2020–2040. We considered 23 diseases that required service utilization at the primary care level and encompass 63% of the total burden of diseases in the Philippines^39^ (Supplement 1). For each of these diseases, the health facility development plan provided estimates on population size, incidence or prevalence rates, number of anticipated primary care consultations, and equipment needed in 81 provinces and 34 highly urbanized cities between 2020 and 2040, which became the basis for service utilization numbers. We converted the service utilization numbers to estimates of the health workforce required for primary care according to (1) which diseases required participation from various health professionals; (2) which service utilization type was applied; and (3) the workforce productivity norms based on 260 working days per year, 6–8 working hours per day excluding weekends and holidays, and time consumption per service episode according to cadre, using the activity standards determined during the DOH’s pilot implementation of the WISN approach in selected regions.^24^ Details of the methodology were further described in the facility development plan.^38^ A complete enumeration of these assumptions together with the corresponding mathematical formulas is also available in Supplement 2, a spreadsheet that other policy makers and program managers could adapt to support their respective health workforce planning processes. As an alternative to target densities, the tool could calculate the future number of health professionals required for the optimal delivery of primary care services beyond 2025.

**FIGURE 5:**
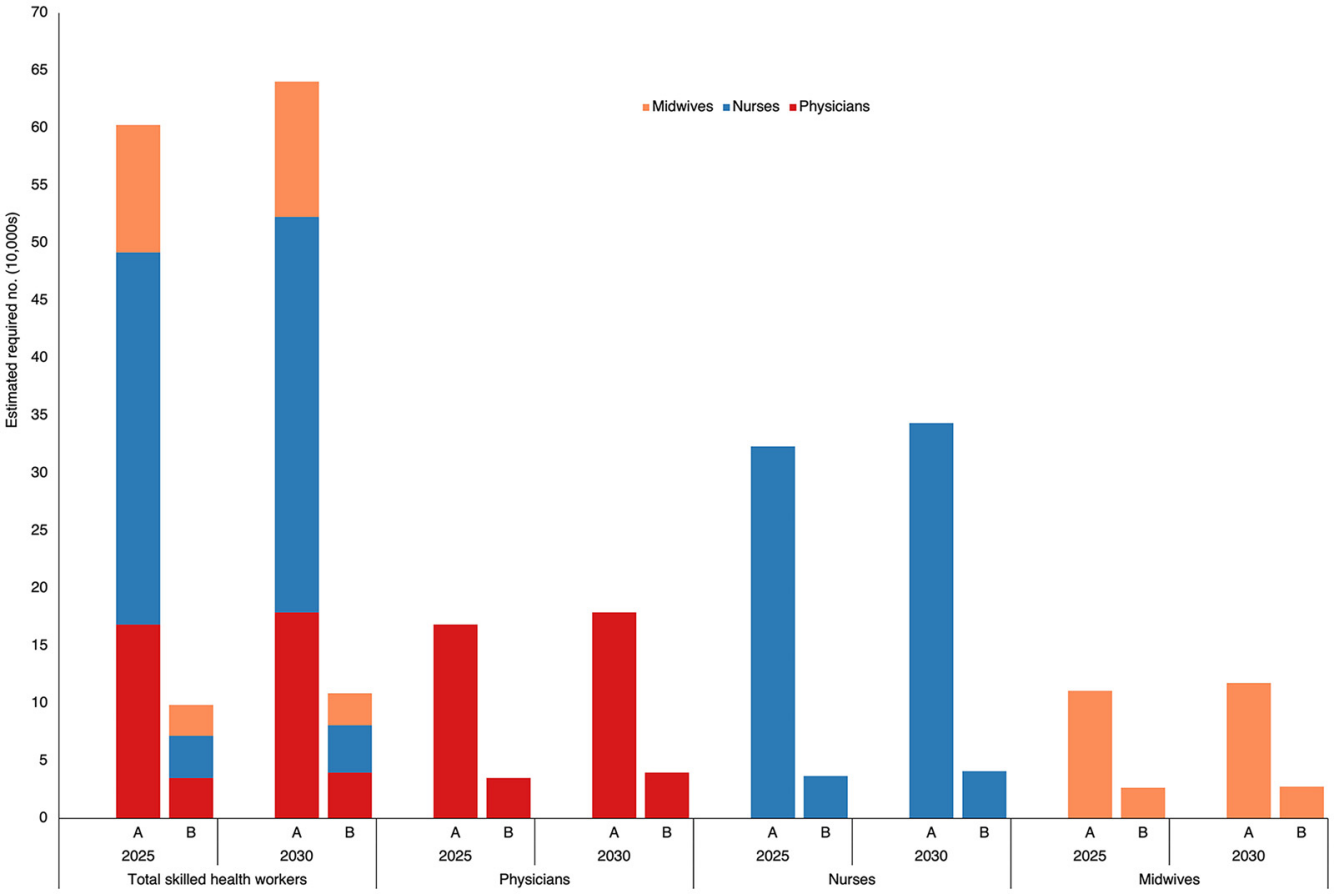
Estimated Number of Skilled Health Workers Required in 2025 and 2030 in the Philippines to Meet the Recommended Density for Universal Health Coverage (A) or the Projected Need for Primary Care Services Based on Epidemiological and Sociodemographic Factors, Service Utilization, and Activity Standards (B)

The estimated health workforce requirements differed depending on the approach: (1) using the target densities to achieve UHC, and (2) the health needs approach to estimate demand for primary care. In Figure 5, we visualized the different estimates for skilled health workers using both approaches for 2025 and 2030. Across the 3 health professions that comprised skilled health workers, the estimated requirements using the benchmark densities were significantly higher than the estimates that considered epidemiological and sociodemographic factors. The lower estimates resulting from the second approach could potentially be due to its focus on primary care services, while the estimates using the target densities were probably higher because it considered not only primary care but also all levels of care in the context of achieving UHC. It was not our objective in this article to determine which methodology was more accurate as either approach could have overestimated or underestimated the numbers in the absence of a gold standard for assessing health workforce requirements. However, this divergence could not have been known had we relied on the ratio-based projections alone. We advocate for using a combination of approaches where policy makers could make decisions based on a breadth of information rather than relying on easy answers drawn from singular indicators. For the development of the health workforce master plan, the estimated health workforce requirement to meet the need for primary care services provided more realistic targets for the government.

The estimated health workforce requirement to meet the need for primary care services provided more realistic targets for the government.

### Projection Trend Analysis Guided Policy Implication Discussions

The DOH expanded participation in the cocreation process through the broader HRHN^20^ and sought input from the health professional associations to interpret the trends from our analysis and discuss policy interventions. We derived the following observations and recommendations.

#### Meeting Current and Future Population Demand for Health Services

The Philippines faces a unique situation of having an abundance of nurses but not enough nursing jobs in the domestic health care sector and poor nursing working conditions that motivate nurses to seek employment overseas.^40^ Without satisfactory working conditions in the public or private sectors, there is a missed opportunity to retain more nurses to serve in the domestic health care system. Policies should consider managing nurse migration so that the countries that receive migrant nurses from the Philippines would also provide incentives to domestic training institutions to ensure the quality of nursing education, as well as to hospitals to support the creation of satisfactory working conditions, including decent compensation and job security.^34^^,^^40^

To meet the recommended benchmark density for physicians, future production of physicians should accelerate at a rate that would outpace the growth of the Philippine population. Our calculations indicated that the Philippines would need to produce the unrealistic target of 65,803 new physicians by 2025, which would already be 78% of the total number of physicians in 2020 (Figure 3). Planning would be more feasible if it also considered the lengthy time to complete physician training. Other cadres in excess, such as nurses, may be tapped through task shifting to perform selected functions in place of physicians in the delivery of primary care services.

To help bridge the gaps in demand for physicians, other cadres in excess, such as nurses, may be tapped through task shifting to perform selected functions in place of physicians in the delivery of primary care services.

Expanded government-funded scholarships should first prioritize physicians, then midwives and dentists whose densities will decline, as well as occupational therapists who had the lowest density among the health professions. However, representatives from the DOH also expressed the need to augment government funding for scholarships with private-sector funding consistent with a whole-of-society approach. These public-private partnerships for scholarships could support training institutions in rural areas with critical shortages and prioritize students from those areas. Evidence has shown that graduates were more likely to practice in areas where they trained and/or come from.^41^ Government regulation of health professionals’ education must also ensure quality assurance to increase completion rates of trainees and successful licensing of graduates as these would be better indicators than the number of graduates in evaluating institutional performance. Recommendations in the master plan included the establishment of more training programs in midwifery, dentistry, and medicine, as well as supporting shorter training programs (e.g., 2-year midwifery programs) or programs with less educational requirements to produce cadres that could augment service delivery typically provided by health professions in short supply (e.g., dental hygienists or dental technologists who could provide oral care in the absence of dentists).^12^

Finally, benchmarks for health professions other than physicians, nurses, and midwives must be explored to provide guidance to countries because these cadres also play important roles in the delivery of primary care services. Although target densities may not always be sensitive in capturing health needs as shown in our analysis, they would be useful in comparing the health workforce situation across countries and subnational levels. The use of benchmark densities as a reference should be complemented by other approaches that consider other factors such as the burden of disease to determine needs more accurately.

#### Achieving an Appropriate Mix of Cadres of the Health Workforce

Combining physicians, nurses, and midwives under the category of skilled health workers created an illusion that the Philippines had surpassed the benchmark of 44.5 workers per 10,000 people to achieve UHC and the SDGs. In actuality, there was a mismatch in the mix of skilled health workers based on their individual densities. The surplus of nurses presents an opportunity to address the shortage of physicians in rural areas, as well as the projected decline in the density of midwives, through task shifting. By enabling policies at the national level that formalize task shifting, nurses could be credentialed, as demonstrated in other settings,^42^ to manage the course of selected diseases, instead of relying only on physicians. Successful task shifting requires enhanced nursing education so that graduates would be prepared to take on additional functions in primary care that would also move away from a traditional physician-centric approach. The mix of cadres could vary at subnational levels, such as in the case of the MIMAROPA region (Figure 4) where even nurses were below the target density. However, midwives exceeded the threshold in this region and therefore could also be credentialed to assume selected functions in place of nurses and physicians. Institutionalizing a legal mechanism for task shifting, however, would be a political process in which the associations of health professions would be protective of their scopes of practice. The HRHN could serve as an important platform for engagement to find common ground among the various health professional associations.

#### Distributing the Health Workforce Equitably To Serve in Areas With the Most Need

Although the Philippines has an extensive health workforce deployment program managed by the DOH, deployment should aim for an appropriate combination of cadres based on varying needs at regional levels. The difference in the densities of skilled health workers between the highly urban National Capital Region and the rural island group region of MIMAROPA clearly indicated inequity in health workforce distribution. Stakeholders agreed on the need to expand existing deployment programs of health professionals, including physicians,^43^ to areas with health workforce shortages. In addition to physicians, deployment programs should also take advantage of the abundance of nurses by offering incentives to attract more nurses to participate in the deployment program and serve in critical areas, such as MIMAROPA. For the COVID-19 pandemic, our analysis of health workforce data also provided guidance to the government on the implementation of the policy for emergency hiring of additional health workers to serve in the health care system.^44^ However, deployment or emergency hiring would only be a temporary solution to health workforce shortages, especially in rural areas. The scholarship program must have an equity dimension to prioritize applicants from regions with the lowest densities of health professions with the expectation that scholars would serve in these areas after securing their license to practice. One master plan recommendation was to increase government investments in public training institutions and expand partnerships with private institutions located in regions with critical shortages that could potentially help increase the retention of health professionals in these areas.^12^ Anecdotal reports received by the DOH also suggested that many health workers in the private sector transferred to the public health sector as soon as vacant positions became available partly because of the disparity in the compensation between the 2 sectors. During the pandemic, this practice resulted in the closure of some private hospitals or downgrading of the classification of private health facilities. To address the importance of the private sector’s role, another master plan recommendation supported financing private health facilities through the national social health insurance system to incentivize health professionals in the private sector to continue serving in critical areas.^12^

Health workforce deployment should aim for an appropriate combination of cadres based on varying needs at regional levels.

## LESSONS ON COCREATION

Research-to-policy collaborations in the Philippines have often been driven by the linear model of knowledge translation. Cocreation provided a mechanism for policy makers and researchers to move closer to a dynamic process that shared power and nurtured relationships to deliver policy-relevant research and jointly addressed health workforce challenges. Such a relationship would also open opportunities for further cocreation to address challenges beyond those related to the health workforce.

In summary, our cocreation process began with a group of stakeholders (i.e., the research team) taking the initiative to explore areas for collaboration with the policy makers as other stakeholders eventually participated in the process. Cocreation occurred through a formal institutional agreement among the partnering institutions with support from top leadership. The ability to identify a financing instrument and mobilize it to support the work was essential. Key factors for success included pooling resources and expertise by using shared workspace that fostered frequent interactions in an environment characterized by open communication and agile coordination of efforts. Through the constant exchange of ideas, researchers learned about policy making while the policy makers appreciated the rigors of research methods. Researchers and policy makers shared power in decision making and jointly identified the priority questions and the suitable methodology to address these questions. The DOH team led the effort to collate the data, and the research team led the analysis of data and trained junior DOH staff on research skills. Both researchers and policy makers interpreted the implications of the results while also expanding the discussions to formulate strategies that included other stakeholders, such as health professional associations. The research output was developed in a format appreciated by the policy makers and used to develop the health workforce master plan for the Philippines. The DOH team was involved in the entire process of evidence generation and interpretation in contrast to the usual practice of conventional knowledge translation where the policy makers wait for the research output.

Our cocreation model was suitable for a health workforce planning process at the meso- and macrolevels of the health care ecosystem^45^ and in the context of developing a national plan for the Philippines. An expansive cocreation model could have also addressed the microlevel through participation from other health workers, patients, and/or community members. We opted for a cocreation process that prioritized the stakeholders in the research and policy arenas in consideration of the limitations in resources and time. We encourage policy makers, program managers, researchers, and other actors in the health care system to consider a cocreation model that is suitable for their goals, as long as the overall aim of providing solutions and achieving societal impact^15^ is maintained. The ideal cocreation model would require involvement from all levels of the health care system with an orientation to be as inclusive of all voices as possible.^46^ However, an expanded cocreation process must also consider how to manage potentially conflicting views from various stakeholders. Future research in this area could investigate what configurations of cocreation have proven successful in addressing health care system challenges. We summarized the lessons from our cocreation process by comparing it with the linear process of research translation according to the phases of a research cycle in Table 2.

**TABLE 2.** Differences Between the Linear Research Translation Model and the Cocreation Model Used by the Philippine Department of Health in the Health Workforce Planning Process

**Knowledge-to-Action Cycle**	**Linear Research Translation**	**Cocreation**
Initiation of process	Policy makers open call for proposals; researchers submit a bid	Researchers approach policy makers
Areas for collaboration	Policy makers set the agenda; researchers align with the agenda	Researchers and policy makers explore topics of mutual interest
Institutional partnering arrangement	Policy makers contract the researchers OR researchers secure a grant and later involve the policymakers	Researchers and policy makers pool resources (e.g., share the same workspace) and formalize the partnership (e.g., through a memorandum of agreement)
Policy or research questions	Either the researchers OR the policy makers formulate the questions	Researchers and policy makers formulate the questions together
Methodology or approach	Researchers determine the methodology	Researchers propose the methodology in consultation with policy makers
Data access and collection	Researchers collect the data	Researchers and policy makers engage partners in government to secure access to data and perform data collection together
Analysis of information	Researchers perform the analysis	Researchers lead the analysis while policy makers are oriented about the approaches
Policy implications	Policy makers receive the report from researchers who make policy recommendations	Researchers and policy makers interpret the findings together; other stakeholders are consulted
Moving to action	Policy makers implement the strategies/policies	Policy makers and researchers hold dialogues with other stakeholders on strategies/policies, including the roles for each

The ideal cocreation model would require involvement from all levels of the health care system and be as inclusive of all voices as possible.

### Limitations

Our analysis of health workforce availability was limited by using licensed health professionals as a proxy count for the number of health professionals practicing in the Philippines. Because some health professionals licensed in the Philippines actually practice overseas, this likely led to an overestimation of the number of health professionals working in the country. Other health professionals may also be licensed but not working in the health care system. However, these data are not captured by the PRC database, which was the basis of our analysis. Our projection studies combined health professionals regardless of public or private practice as the PRC database only captured the health professional’s residence and not the type of health facility where they practice. The master plan^12^ included a recommendation to establish an integrated health workforce information system that would harmonize data from various sources and address existing data gaps by capturing data on areas of practice, vacancy rates in the public and private sectors, migration, retirement, and deaths for a more comprehensive assessment of health workforce situation. With such a system in place, future projection studies could be more granular and agile without having to request datasets from different government agencies every time health workforce planning is conducted. Future studies should complement our focus on quantity, skill mix, and distribution with investigations into the equally important issues of competence, productivity, and satisfactory working conditions, including the push and pull factors in the labor market that influence health workforce motivation and retention, as well as the effects of global developments such as pandemics and climate change.

## CONCLUSIONS

We described our cocreation process to inform the development of the National Human Resources for Health Master Plan, which the Philippine government published in 2021. Findings from the analysis of the adequacy of the health workforce became a major component of Chapter 2 (Situation, Results Framework, and Strategies), while our estimates of future health workforce requirements and recommendations contributed to Chapter 6 (Health Education Strengthening and Regulation). Our methodology and results also became an annex to the master plan.

Ministries of health, especially in low- and middle-income countries, are often—understandably—preoccupied with addressing the most pressing problems of the present and may overlook the need to plan for the future of health care systems.^47^ We have shown that cocreation facilitated the use of data analysis in planning by embedding a team of researchers in the policy-making space and involving stakeholders in the process. Given the complexity of health workforce challenges, there is no single planning method that would provide all the answers. Our cocreation process, methods and analysis, and discussion of policy implications may offer guidance to policy makers, researchers, and other stakeholders on how they may proceed with their respective health workforce planning.

To achieve UHC and the SDGs, health workforce planning should not be a single event but rather a process of continuing recalibration in response to dynamic scenarios and emerging opportunities and threats, including unprecedented events like the COVID-19 pandemic. Planning effectively for the health workforce requires responding to the challenges of the present with a constant gaze toward the future. Government agencies responsible for national health workforce development may do well to invest in strengthening internal capacities in evidence-informed policy making and advance the idea of cocreation with research institutions and other stakeholders to shape health care systems that are responsive and resilient both during normal times and during crises.

## Supplementary Material

GHSP-D-22-00176-Supplement_1.pdf

GHSP-D-22-00176-Supplement_2.xlsx

## Notes

**Peer Reviewed**

**First published online**: November 14, 2022.
