# Supplementary material for: Cocreation in Health Workforce Planning to Shape the Future of the Health Care System in the Philippines
Source: Glob Health Sci Pract. 2022 Dec 21;10(6):e2200176. doi: 10.9745/GHSP-D-22-00176 (PMC9771466; doi:10.9745/GHSP-D-22-00176)
Supplement: GHSP-D-22-00176-Supplement_1.pdf [file GHSP-D-22-00176-Supplement_1.pdf]

**Supplement to**: Liwanag HJ, Uy J, Politico MR, et al. Cocreation in health workforce planning to shape the future of the health care system in the Philippines. *Glob Health Sci Pract*. 2022;10(6):e2200176. https://doi.org/10.9745/GHSP-D-22-00176

## Supplement 1

List of the 23 causes of morbidity and mortality we considered for estimating demand for primary care based on the Global Burden of Disease. [DALYs: disability-adjusted life year]

| Cause | Share of total DALYs | Cause | Share of total DALYs |
|---|---|---|---|
| **A. Noncommunicable Diseases** | | | |
| Ischemic heart disease | 7.93% | Asthma | 1.92% |
| Stroke | 6.06% | Hypertension (Hypertensive heart disease) | 1.74% |
| Diabetes mellitus | 3.92% | Breast cancer | 1.02% |
| Chronic kidney disease | 3.31% | Lung cancer | 1.01% |
| Chronic obstructive pulmonary disease | 2.90% | Colon and rectum cancer | 0.75% |
| **B. Maternal and Child** | | | |
| Neonatal disorders | 7.54% | Malnutrition | 0.49% |
| Congenital birth defects | 3.40% | Maternal disorders | 0.38% |
| **C. Infectious Diseases** | | | |
| Lower respiratory infections | 7.08% | HIV/AIDS | 0.80% |
| Tuberculosis | 3.40% | Dengue | 0.70% |
| Diarrheal diseases | 1.63% | | |
| **D. Mental Health and Injuries** | | | |
| Low back pain | 2.93% | Depressive disorders | 1.13% |
| Road injuries | 1.98% | Drug use disorders | 0.53% |
